# Imaging Inelastic Fracture Processes in Biomimetic Nanocomposites and Nacre by Laser Speckle for Better Toughness

**DOI:** 10.1002/advs.201700635

**Published:** 2017-12-18

**Authors:** Tuukka Verho, Pasi Karppinen, André H. Gröschel, Olli Ikkala

**Affiliations:** ^1^ Molecular Materials Department of Applied Physics Aalto University FI‐02150 Espoo Finland; ^2^ ProtoRhino Ltd Betonimiehenkuja 5C FI‐02150 Espoo Finland; ^3^ Department of Physical Chemistry and Centre for Nanointegration Duisburg‐Essen (CENIDE) University of Duisburg‐Essen D‐45127 Essen Germany

**Keywords:** biomimetics, mechanical properties, nanocomposites, process zone, toughness

## Abstract

Mollusk nacre is a prototypical biological inorganic–organic composite that combines high toughness, stiffness, and strength by its brick‐and‐mortar microstructure, which has inspired several synthetic mimics. Its remarkable fracture toughness relies on inelastic deformations at the process zone at the crack tip that dissolve stress concentrations and stop cracks. The micrometer‐scale structure allows resolving the size and shape of the process zone to understand the fracture processes. However, for better scalability, nacre‐mimetic nanocomposites with aligned inorganic or graphene nanosheets are extensively pursued, to avoid the packing problems of mesoscale sheets like in nacre or slow in situ biomineralization. This calls for novel methods to explore the process zone of biomimetic nanocomposites. Here the fracture of nacre and nacre‐inspired clay/polymer nanocomposite is explored using laser speckle imaging that reveals the process zone even in absence of changes in optical scattering. To demonstrate the diagnostic value, compared to nacre, the nacre‐inspired nanocomposite develops a process zone more abruptly with macroscopic crack deflection shown by a flattened process zone. In situ scanning electron microscopy suggests similar toughening mechanisms in nanocomposite and nacre. These new insights guide the design of nacre‐inspired nanocomposites toward better mechanical properties to reach the level of synergy of their biological model.

Natural composite materials, particularly nacre from mollusk shells, have inspired new kinds of man‐made inorganic/organic composites that aim at structural applications.^1^, ^2^, ^3^, ^4^ Nacre and other mineralized natural composites like bone, enamel, and the stomatopod dactyl hammer exploit the stiffness and hardness of ceramics while eliminating their inherent brittleness with ingenious hierarchical microarchitectures.^5^, ^6^, ^7^, ^8^, ^9^, ^10^, ^11^, ^12^, ^13^, ^14^ Synthetic mimics have replicated the properties of nacre with some success.^15^, ^16^, ^17^, ^18^, ^19^, ^20^, ^21^, ^22^, ^23^ However, the toughening mechanisms in biological and bioinspired composites are diverse and quite unlike those in traditional structural materials such as metals. Fracture in these highly inhomogeneous and anisotropic microstructured composites poses challenges for theoretical description.^24^, ^25^ In the study of fracture, of particular interest is the process zone ahead of the crack, where energy dissipating inelastic deformation takes place, spreading out the stress concentration.^24^ Recently, we made a significant advance in studying fracture in nacre‐inspired clay/polymer nanocomposites by reporting on a method to fabricate laminated bulk plates from nanocomposite films.^26^ This vertical scale‐up approach made possible the first proper fracture characterization of a self‐assembled nacre‐mimetic nanocomposite thanks to the millimeter‐to‐centimeter scale sample thickness, revealing a promising fracture toughness of *K*_Ic_ = 3.4 MPa m^1/2^. However, further improvements in toughness require deeper characterization and understanding of the fracture processes. Here, we adopt a novel crack tip imaging method that allows imaging the local damage processes ahead of a crack in red abalone nacre and a nacre‐mimetic clay/polymer nanocomposite that consists of self‐assembled clay nanoplatelets in a polyvinyl alcohol matrix. With laser speckle imaging, we trace the real‐time evolution of the process zone and crack propagation, and use in situ scanning electron microscopy (SEM) for complementary microscopic insight. The process zone is initially similar in size in both materials, but in the biomimetic nanocomposite, it becomes flattened as a result of macroscopic crack deflection. This has implications for fracture toughness determination, and gives direction for further development by suggesting that improve adhesion between the clay nanoplatelets is needed.

The toughness of nacre relies on its microstructure that consists of roughly 500 nm thick aragonite platelets separated by thin organic layers (**Figure** **1**a,b) that can dissipatively slide against each other under mechanical loads. This microstructure and capability to inelastic deformation through platelet sliding is being replicated in synthetic mimics such as self‐assembled clay/polymer nanocomposites (Figure 1a,c). In nacre and other materials that can deform through sliding between brittle components, a number of toughening mechanisms have been identified, including crack deflection, uncracked filament bridging, and constrained microcracking.^24^ These inelastic processes take place within the fracture process zone at the crack tip, prior to and during crack propagation. In nacre, the process zone has been carefully studied, taking advantage of the optically scattering gaps that open between the platelets as a result of platelet sliding, or using atomic force microscopy.^8^, ^12^, ^13^, ^14^ However, a general way to observe the process zone in nacre‐mimetic materials is lacking, because for instance clay/polymer nanocomposites do not have scattering features to facilitate optical detection.

**Figure 1:**
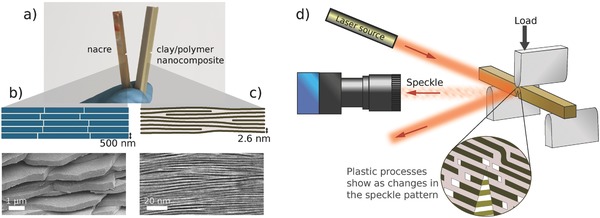
The structures of the biological and biomimetic composites, and the laser speckle imaging method. a) Notched fracture test beams of abalone nacre and nacre‐inspired clay/polymer nanocomposite. b) A scheme and an SEM microscopic image of the microstructure of abalone nacre. c) A scheme and a cross‐sectional TEM microscopic image of the nanostructure of the clay/polymer nanocomposite. d) A scheme of the laser speckle imaging setup.

Figure 1d shows our setup to image the process zone in nacre and nacre‐mimetic composites with laser speckle. Microscopic inhomogeneities on the sample surface cause a random speckle pattern when illuminated with a laser, allowing the detection of very small changes on the surface topography. The inelastic deformation within the process zone is easily detectable in this manner, because it consists of discrete and fast events (such as opening of microcracks) that cause significant local changes in topography that alter the speckle pattern (see Video S1 in the Supporting Information). Elastic deformation, in contrast, is homogenous and gradual, and alters the speckle pattern at a much slower rate. The process zone can be identified from a high‐speed video of the speckle pattern by highlighting pixels undergoing fast intensity changes.

Previously, digital image correlation (DIC) based on white light or laser speckle has been used to measure surface displacement fields during mechanical testing.^27^, ^28^, ^29^ However, in addition to requiring more calibration, pretreatment, and postprocessing, DIC is unable to give direct information on the process zone like the present method, as it is indifferent to the physical process causing the deformation, and only measures displacements. It has been reported that laser speckle patterns can be used to measure plastic strain in metals by analyzing the spectral intensity of the reflected beam.^30^ As that method cannot be directly used for imaging, we chose the simpler approach of detecting intensity changes in the speckle image. A similar approach has previously been applied to measuring blood flow,^31^ but according to our knowledge, it has not been used for fracture process zone imaging before.

**Figure** **2** summarizes the laser speckle imaging of hydrated red abalone (*Haliotis rufescens*) nacre (also shown in Video S2, Supporting Information). Approximately 1.5 mm wide (width and height) beams with a sharp precrack were tested in a single edge notched bending (SEB) experiment. The series of four snapshots in Figure 2a shows that first signs of process zone formation can be seen soon after the mechanical response becomes nonlinear. A time series plot in Figure 2b shows the development of the horizontal process zone profile (signal profile along a horizontal band crossing the process zone). The plot shows a short development phase during which the process zone attains its final width of ≈0.4 mm. Previously, the process zone of nacre has been studied by observing the whitening zone at the crack tip that provides clear indication of platelet sliding taking place.^8^, ^12^ Therefore, the speckle imaging can be compared against the traditional method for verification. We found that the speckle signal overlaps with the whitening zone very well (Figure S1, Supporting Information). To rule out the possibility that the increased scattering in the process zone would create a false laser speckle signal, we measured a sample coated with a sputtered Au layer to eliminate scattering from inside the specimen; a signal could still be observed (Figure S1, Supporting Information).

**Figure 2:**
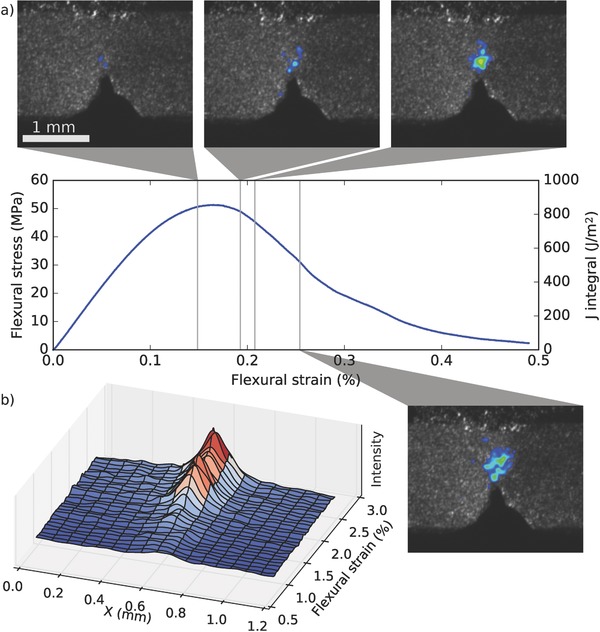
Fracture and process zone formation in red abalone nacre. a) Four snapshots of the laser speckle measurement showing the gradual development of the process zone during a SEB test of hydrated nacre. b) A time series of the approximate cross section of the speckle difference signal. The profiles are taken over a horizontal band over the process zone. The plot shows an onset of the signal close to 1.5% strain and a more rapid increase around 2.0% strain, in agreement with the snapshots in panel (a).

After confirming that the speckle imaging method correctly detects the process zone in nacre, we investigated our nacre‐inspired clay/polyvinyl alcohol nanocomposite to study its crack tip zone before and during crack propagation. Unlike nacre, process zone formation in nacre‐mimetic materials has not been carefully studied. In particular, fracture characterization of nacre‐mimetic clay/polymer nanocomposites has not been possible due to the difficulties in producing self‐assembled samples thicker than films. Here, we follow our recently published approach to produce three millimeter thick clay/polymer nanocomposite samples by combining evaporation‐induced self‐assembly with lamination.^26^

**Figure** **3**a shows a series of six snapshots of the process zone on the nacre‐inspired clay/polymer nanocomposite at various stages of progression (see also Video S3 in the Supporting Information). Initially, inelastic deformation takes place within a roughly circular region with a diameter of roughly 0.5 mm, but eventually deforms into a flattened horizontal stripe. This reflects the material's strong tendency to deflect cracks, as shown by Morits et al.^26^ The crack prefers to deflect in the approximate direction of the clay platelets rather than advance perpendicular to them (“face‐on”). As shown by the time series of the process zone profile in Figure 3b, the formation of the process zone is less gradual than in nacre. In fact the appearance of the process zone is accompanied by a briefly larger signal intensity indicating more inelastic deformation, also shown in snapshot 2. After the deflected crack initiates, it grows both left and right, sometimes showing much more activity on one side or the other, as shown by snapshots 4–6 and Figure 3b. A similar development is shown in Video S4 (Supporting Information), showing a different measurement.

**Figure 3:**
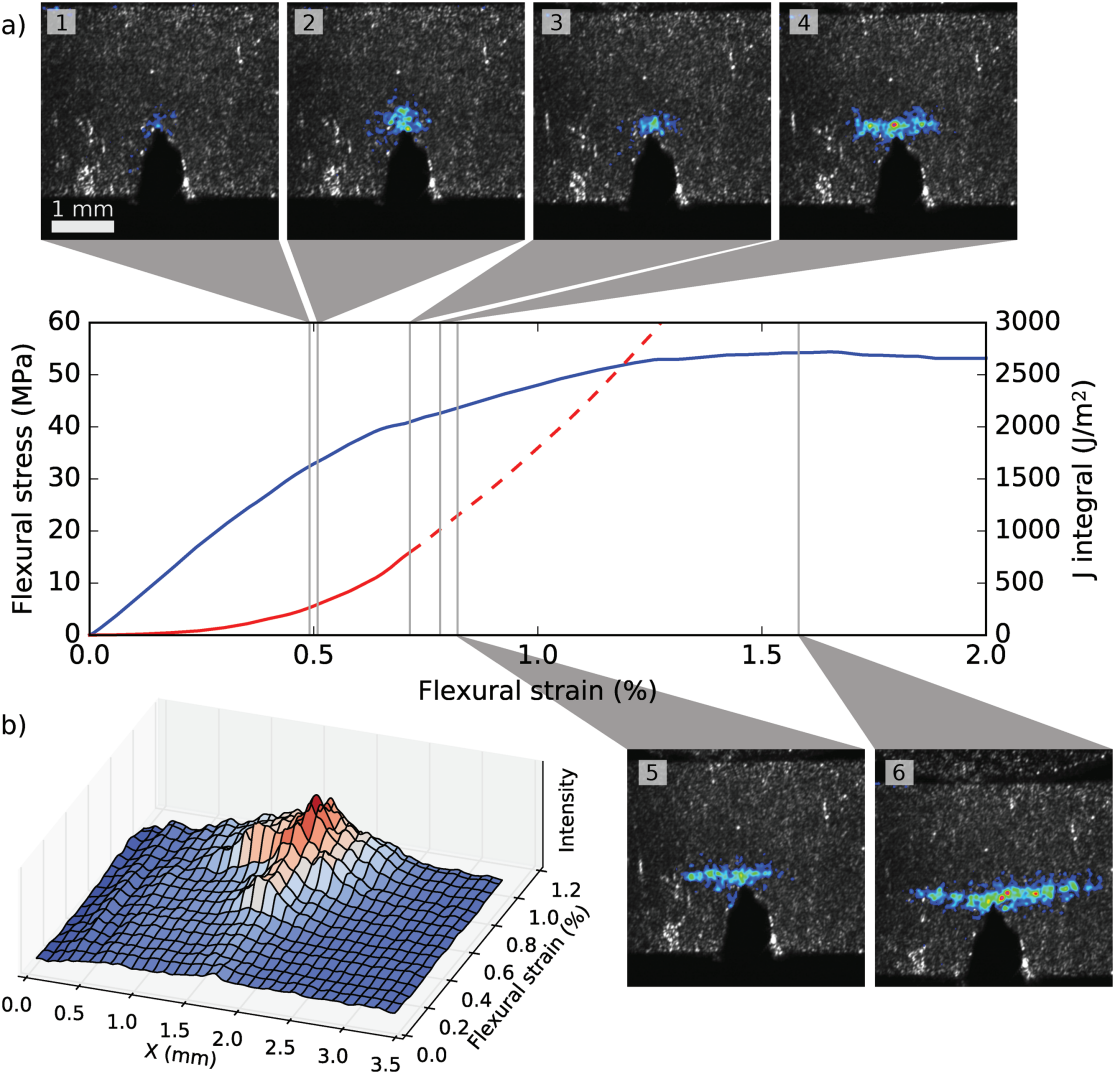
Laser speckle imaging during single edge notched bending experiment for biomimetic clay/polyvinyl alcohol nanocomposite. a) Six snapshots showing the process zone development and a plot of stress and the *J* integral as a function of strain. The nominal value of the *J* integral after deflected crack initiation is shown with a dashed curve. b) The evolution of the horizontal signal profile. At 0.5% strain, the process zone forms rather abruptly and subsequently diminishes slightly in size (snapshots 2 and 3). After some time, a deflected crack forms (snapshot 4), expressing as widening of the process zone profile in panel (b).

Figure 3a also plots the *J* integral (see the Supporting Information) as a function of flexural strain. As the crack becomes deflected at ≈0.8% strain and the crack initiation does not lead to imminent fracture, the nominal value of the *J* integral grows very large. However, the data after the initiation of the deflected crack growth can hardly be regarded valid, as the *J* integral is defined for a straight crack. Therefore, without imaging the crack tip, it can be hard to exactly define the ultimate value for the *J* integral. Importantly, with laser speckle imaging, we can pinpoint the moment of crack deflection, which allows an accurate determination of the critical *J* integral. For the experiment in Figure 3 we obtain a value of ≈800 J m^−2^, corresponding to a fracture toughness of *K*_Ic_ = 4.7 MPa m^1/2^, which is slightly larger than measured before.^26^

The size of the process zone has been linked to the flaw tolerance of the material, i.e., the size of a defect that is required to significantly reduce the strength of a specimen due to crack nucleation.^32^ Here, the process zone widths in nacre and the nanocomposite were comparable, although the nanocomposite seemed to have a slightly larger zone (0.5 mm) than nacre (0.4 mm). In the more simple case of isotropic elastic–plastic materials, the size of the plastic zone *r*_p_ has been shown^25^ to depend on the stress intensity factor *K*_I_ and the yield stress σ_Y_

(1) $$r_{p}\sim \frac{K_{I}^{2}}{\sigma_{Y}^{2}}$$

The flexural yield stress of nacre and the nanocomposite is similar of the order of 200 MPa, so it is somewhat unexpected that the nanocomposite does not have a smaller process zone than nacre despite its lower *K*_Ic_. This could be explained by crack tip shielding processes (discussed below) that do not take place in the process zone but still increase toughness (not considered by Equation (1)). Also, the anisotropy of the materials can play a role. For the nanocomposite, the constant of the proportionality in Equation (1) is 0.9, which is ten times larger than the value 1/3π predicted by the Irwin approach for crack tip plasticity.^25^ This result indicates that further research is needed to understand the relationship between process zone size, flaw tolerance, and fracture toughness in the anisotropic nacre‐inspired materials.

In biological materials like nacre, a number of toughening mechanisms have been identified, including crack deflection, uncracked filament bridging, and constrained microcracking.^24^ Deflection and bridging are considered extrinsic (crack tip shielding) mechanisms that mostly act behind the crack tip, reducing the stress intensity at the tip. Microcracking, on the other hand, takes place ahead of the crack tip within the process zone, and is termed intrinsic toughening. If microcracking does not lead to advancement of the main crack, it can create a cloud of microcracks that effectively cause inelastic deformation, leading to resistance to crack propagation.

While the toughening mechanisms in nacre are relatively well known, it is unclear to what extent similar mechanisms are activated in the clay/polymer nanocomposite. To find out, we performed in situ SEB measurements in SEM. Compared to post mortem SEM imaging, in situ microscopy gives a fuller picture of the inelastic damage taking place, as the progression of the damage and all cracks are much more clearly visible and open in the stressed state. As shown in **Figure** **4**, various forms of deflection, crack bridging, and microcracking can be observed. Deflection happens early in the experiment, either as soon as the main crack nucleated or soon after some initial straight propagation (Figure 4a). In the latter cases, we witnessed a horizontal crack forming ahead of the straight crack, and their subsequent merging as the straight crack reached the horizontal crack (Figure S2a, Supporting Information). Crack bridging and microcracking could not always be differentiated, as microcracks typically formed ahead and around the progressing crack, and then some of them clearly became extensions of the main crack, even though a filament still separated them (Figure 4b–d). In the clay/polymer nanocomposite, the crack often grows by the initiation of a crack ahead of the tip of the main crack and the subsequent coalescence of the microcrack with the main crack. This makes it sometimes difficult to define to extent of the main crack—which helps to diffuse the stress concentration and allows the irreversible deformation to occur over a larger area. In some cases, a branch forms in the deflected crack that grows back toward the center of the beam where the bending moment is largest, creating a zigzag‐shaped crack path (see Figure 4e). More in situ SEM images are shown in Figure S2 (Supporting Information).

**Figure 4:**
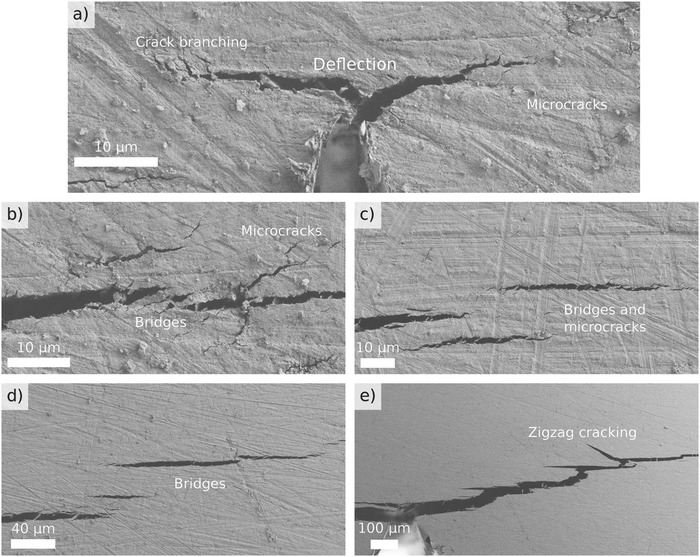
In situ scanning electron microscopic images of a clay/polymer nanocomposite in different stages of a SEB test. a) Deflected crack initiated from the precrack made with a razor blade. b) Nucleation of microcracks, some of which subsequently merge with the main crack. c) Larger microcracks within the process zone. d) Thick bridges between microcracks that appear to be extensions of the main crack. e) A crack branch grows back toward the center, leading to zigzag cracking.

Unlike intrinsic toughening that takes place ahead of the crack tip, extrinsic mechanisms only act after the crack has nucleated. This leads to an increase in fracture resistance as the crack advances (rising *R*‐curve). It has been proposed that the tortuous crack path in nacre creates points of contact between the crack faces, explaining the observed rising *R*‐curve.^8^ In the laser speckle imaging, signal was detected behind the crack tip in both nacre and the nanocomposite, indicating inelastic deformation caused by crack bridging (Figures 2 and 3; Videos S2–S4, Supporting Information).

Although the in situ SEM shows clear evidence of a process zone where microcracks of various sizes nucleate, the size of the region where they could be observed in SEM is smaller than the process zone according to laser speckle imaging. We hypothesize that the laser speckle method is much more sensitive to small changes than SEM and shows indication of inelastic deformations that take place beneath the surface of the sample. What these deformations might be still remains an open question, but they could be related to the opening of microcracks, or sliding of the clay platelets or stacks of platelets against each other.

The tendency of the clay/polymer nanocomposite to form a flattened process zone and the large number of horizontal cracks nucleating around the tip in in situ SEM images suggest that the resistance of the material to the formation of voids in the matrix between the platelets is comparatively low. This promotes crack deflection, but likely also limits the fracture toughness of the material. To take the toughness of the nacre‐mimetic nanocomposite to a new level, a better adhesion between the clay nanoplatelets is needed. However, the ability of the matrix to undergo plastic shear should be maintained to avoid losing the capability to dissipation. Careful design of molecular interactions is required to tailor a matrix that resists cavitation and desorption but is capable of large plastic shear.

The laser speckle method is used here mostly as a surface imaging technique, although the partial translucency in the materials may bring also a subsurface contribution to the signal. Therefore, the results mostly reflect what takes place close to the surface, which however should be representative of the bulk behavior with reasonable accuracy. Some modification to the observed shape of the process zone can be expected due to the lower stress triaxiality on the surface^25^ and the possible presence of surface defects. However, transmission mode measurement would be a possibility for translucent or thin samples. In that case, the laser passes through the sample and the signal represents the collective contribution of the entire sample thickness. Recently, biomimetic or biobased translucent or transparent materials with colloidal level nanocellulose‐ or nanoclay‐based constituents have been introduced.^33^, ^34^, ^35^, ^36^, ^37^ The laser speckle imaging may present a valuable means to explore the hitherto poorly known fracture processes in colloidal materials.

To conclude, we presented a new laser speckle based fracture process zone imaging method to study nacre‐inspired inorganic/organic composites to better understand fracture and toughness in the materials. Also, the concept allows to better determine the ultimate value of the *J* integral. The process zone in nacre and a nacre‐inspired clay/polymer nanocomposite was studied, and an approximately half‐a‐millimeter sized process zone was found in either material. The process zone in nacre developed gradually, while the nanocomposite showed more abrupt crack tip zone development. Furthermore, macroscopic crack deflection was seen in the laser speckle imaging. In situ SEM was used to identify microscopic toughening mechanisms in the clay/polymer nanocomposite. Similarly to what has been observed in nacre, crack deflection, microcracking, and uncracked ligament bridging were found. The strong tendency to form cracks that grow between the clay nanoplatelets suggests that stronger adhesion between the platelets would be desirable for better fracture resistance, even though the tendency also promotes toughness by causing crack deflection. Based on the promising results with the clay/polymer nanocomposite and nacre, we propose the laser speckle imaging method as a useful fracture imaging method to get deeper insight on the dynamic and instantaneous fracture processes to promote toughness.

## Conflict of Interest

The authors declare no conflict of interest.

## Supporting information

Supplementary

Supplementary

Supplementary

Supplementary

Supplementary

Supplementary
