# Supplementary material for: Imaging Inelastic Fracture Processes in Biomimetic Nanocomposites and Nacre by Laser Speckle for Better Toughness
Source: Adv Sci (Weinh). 2017 Dec 18;5(1):1700635. doi: 10.1002/advs.201700635 (PMC5770684; doi:10.1002/advs.201700635)
Supplement: Supplementary file 1 — Supplementary [file ADVS-5-na-s002.pdf]

Copyright WILEY-VCH Verlag GmbH & Co. KGaA, 69469 Weinheim, Germany, 2017.

ADVANCED SCIENCE Open Access

Supporting Information

for *Adv. Sci.,* DOI: 10.1002/advs.201700635

Imaging Inelastic Fracture Processes in Biomimetic Nanocomposites and Nacre by Laser Speckle for Better Toughness

*Tuukka Verho, Pasi Karppinen, André H. Gröschel, and Olli Ikkala**

Copyright WILEY-VCH Verlag GmbH & Co. KGaA, 69469 Weinheim, Germany, 2016.

Supporting Information

Imaging Inelastic Fracture Processes in Biomimetic Nanocomposites and Nacre by Laser Speckle for Better Toughness

*Tuukka Verho, Pasi Karppinen, André H. Gröschel, Olli Ikkala**

Experimental Section

Preparation of the nacre-mimetic clay/polymer nanocomposite plates: To prepare approximately 70 µm thick self-assembled clay/PVA films, 5 wt% aq. dispersion (stirred with an overhead stirrer for at least 2 d) of montmorillonite clay (cloisite Na+, Southern Clay Products) was added dropwise to 5 wt% aq. solution of PVA (Aldrich, M_w = 85 000–124 000 g mol^{-1}, >99% hydrolyzed) while stirring. The resulting dispersion with 60:40 of clay and PVA by mass was homogenized with an overhead stirrer for at least 4 d. Films were cast by pouring 40 mL of dispersion to 120 × 120 mm Petri dishes and allowed to evaporate for ≈3 d. Thick nanocomposite plates were made by applying 1 wt% PVA solution on the films with a Superlon swipe and carefully placing 50 films one-by-one on top of each other. The stacks were compressed at 1–4 GPa between water absorbing concrete slabs while subjecting them to gradually elevating temperatures to remove water. Hard plates were obtained in 2–3 d by applying an increasing temperature up to 100 °C.

Sample preparation: Nacre-inspired montmorillonite/PVA nanocomposite plates with a thickness of roughly 3 mm were prepared by film casting and laminating as described in earlier work.[1] Approximately 5 mm thick beams were cut from the plate and annealed in 130 ºC for 3 d in order to increase the crystallinity of PVA and remove water. After annealing, the width of the beams was reduced to approximately 3 mm by sanding with silicon carbide paper, and grit sizes increasing progressively to 4000 were used to produce very smooth surfaces. Red abalone nacre (*haliotis rufescens)* from California, USA was obtained in dehydrated state. The shell was hydrated in 3.5% salt water for at least 8 weeks. Test samples were cut from the shell with a Dremel 4000 multitool while periodically cooling down the shell with running water. Silicon carbide paper (with water) was used to shape the samples into rectangular beams with a width and height of 1.5-2.5 mm. For SEB tests, pre-cracks were created by first creating a coarse notch manually with a Dremel cut-off wheel, and then making a sharper tip with diamond wafering blade. Finally, the tip was sharpened with a razor blade.

Mechanical testing: In laser speckle measurements, SEB testing was performed with an Instron 5567 tester with a 1kN load cell in three-point bending. A support span of 30 mm and a flexural strain rate of 2%/min was used. In in-situ SEM, a Deben Microtester with a 200N load cell was used in three-point bending with a support span of 23 mm. In our previous study,[1] we found that the apparent flexural modulus of unnotched nanocomposite beams was sensitive to the aspect ratio of the beam, but we did not detect qualitative differences in the fracture process related to the support span. Therefore, the rather short support span in the

notched specimens in in-situ SEM should not affect the interpretation of the results. Displacement was increased stepwise, and SEM images were taken at constant displacement.
Laser speckle imaging: A DynaMat laser speckle imaging system purchased from ProtoRhino Ltd. was used. The bending sample was illuminated with a 642 nm laser and the speckle pattern was recorded with a high speed camera with a 10.6X macro objective and an aperture smaller than f/16. Clay/polymer nanocomposite samples were sputtered with Au for 1 min to reduce optical transmittance to avoid artefacts caused by refraction and reflection inside the specimen.
Laser speckle image analysis: To implement a robust frequency band filtering method for fast analysis of the laser speckle video data, we compared pixel intensities over a given interval. The magnitude of the difference is mostly affected by frequency components whose period is of the same order as or shorter than the interval. Moreover, to cut off high frequency noise, the analysis was performed to a temporally smoothed intensity applying a moving average over a period of t_{avg}. The speckle difference signal amplitude S is then given by

$$S(x,y,t) = \frac{\left|\tilde{I}(x,y,t) - \tilde{I}(x,y,t-t_{\mathrm{d}})\right|}{\tilde{I}(x,y,t)} \tag{2}$$

where

$$\tilde{I}(t) = \frac{\int_{t-t_{\mathrm{avg}}}^{t} I(t)dt}{t_{\mathrm{avg}}} \tag{3}$$

$I(t)$ being pixel intensity and t_{avg} the smoothing interval. Finally, a spatial Gaussian smoothing with standard deviation of 2 pixels was applied to smooth out spatial fluctuations present because of the nature of the speckle pattern. For visualization, pixels with signal intensity above a noise cutoff level were regarded as a part of the process zone. A video frame rate of 500 s^{-1} and analysis was used with a comparison interval $t_{\mathrm{d}} = 100$ ms and an averaging period of $t_{avg} = 20$ ms (clay/polymer nanocomposite) or $t_{\mathrm{avg}} = 60$ ms (nacre). The parameters were chosen to maximize signal-to-noise ratio. Noise cutoff level for the speckle difference signal was set to a level where most of the noise caused by external vibrations was removed.
SEM imaging: A Zeiss Sigma VP electron microscope was used. Before imaging, samples were sputtered with a thin Au layer for 1 min to increase conductivity.
TEM imaging: Imaging was performed in bright-field mode on a Zeiss CEM 902 electron microscope operated at 80 kV. To visualize the alignment of the 1 nm clay nanoplatelets, ultra-thin sections were cut from the bulk nanocomposite with a Reichert-Jung Ultracut E equipped with a diamond knife.

Calculation of the J integral: The J integral was calculated from the sum $J = J_{el} + J_{pl}$. The elastic part was given by $J_{el} = K_I^2/E'$ where $E' = E/(1-\nu^2)$ and

$$K_I = \frac{P}{B\sqrt{W}} f\left(\frac{a}{W}\right) \tag{S1}$$

where *P* is the applied load, *a* is the length of the pre-crack, *W* is the vertical dimension of the beam (in the direction or crack propagation), *B* is depth of the sample, and *f* is

$$f\left(\frac{a}{W}\right)=\frac{3\frac{S}{W}\sqrt{\frac{a}{W}}}{2\left(1+\frac{2a}{W}\right)\left(1-\frac{a}{W}\right)^{\frac{3}{2}}}\left[1.99-\frac{a}{W}\left(1-\frac{a}{W}\right)\left\{2.15-3.93\left(\frac{a}{W}\right)+2.7\left(\frac{a}{W}\right)^{2}\right\}\right] \quad \text{(S2)}$$

where S is the support span.

The plastic part J_{pl} is calculated from the area under the loading-hypothetical unloading curve A_{pl} as in the ASTM 1820 standard as

$$J_{pl}=\frac{2A_{pl}}{Bb} \quad \text{(S3)}$$

where $b=W-a$. Unloading compliance was taken as equal to the initial compliance.

References

[1] M. Morits, T. Verho, J. Sorvari, V. Liljeström, M. A. Kostiainen, A. H. Gröschel, O. Ikkala, *Adv. Funct. Mater.* **2017**, *27*, 1605378.

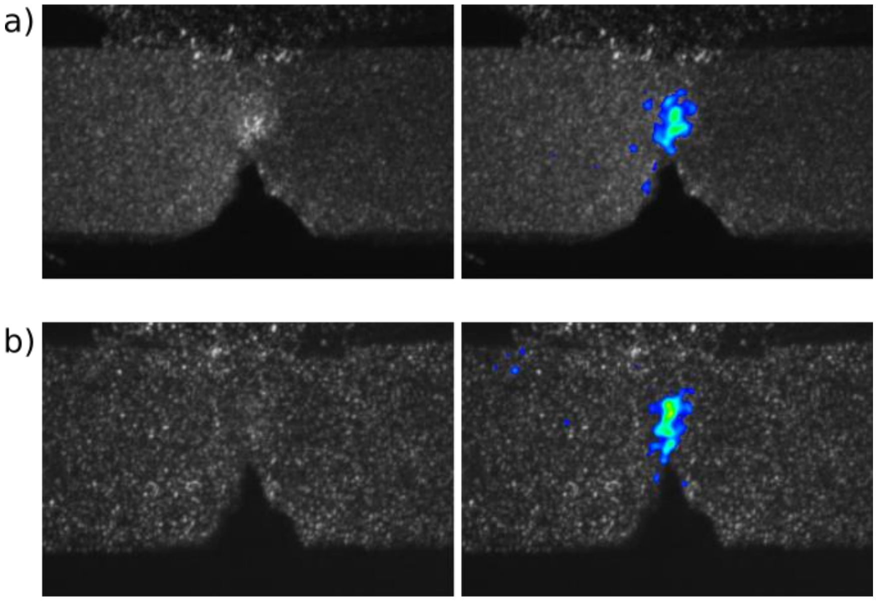

Figure S1. The whitening zone in red abalone nacre compared with the process zone revealed by laser speckle imaging. a) Nacre in laser speckle imaging without a sputtered Au layer. Left and right show the image without and with the detected process zone overlaid, correspondingly. The speckle signal overlaps with the whitened region, which confirms that the speckle is caused by plastic processes (platelet sliding) that also cause the whitening. b) Nacre with a sputtered Au layer. The whitening is masked, but the speckle signal can still be seen.

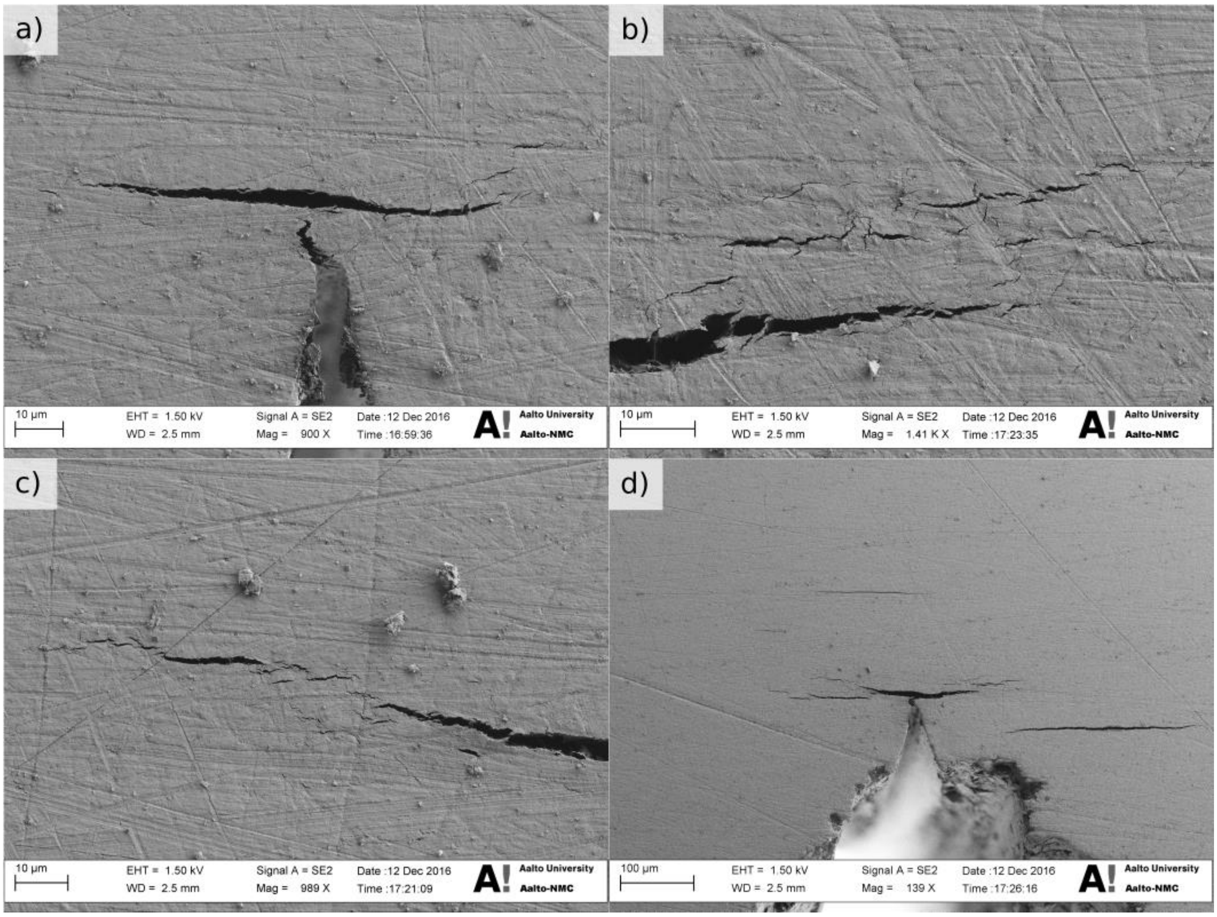

Figure S2. More SEM micrographs of nacre-mimetic clay/polymer nanocomposite in a SEB test. a) Deflection through microcrack formation ahead of the initial straight crack. b) A diffuse tip of the deflected crack showing microcracking and bridging. c) The other tip of the deflected crack. d) New deflected cracks nucleated elsewhere, also behind the original tip. This is likely to be a result of the toughening shown in b) and c).

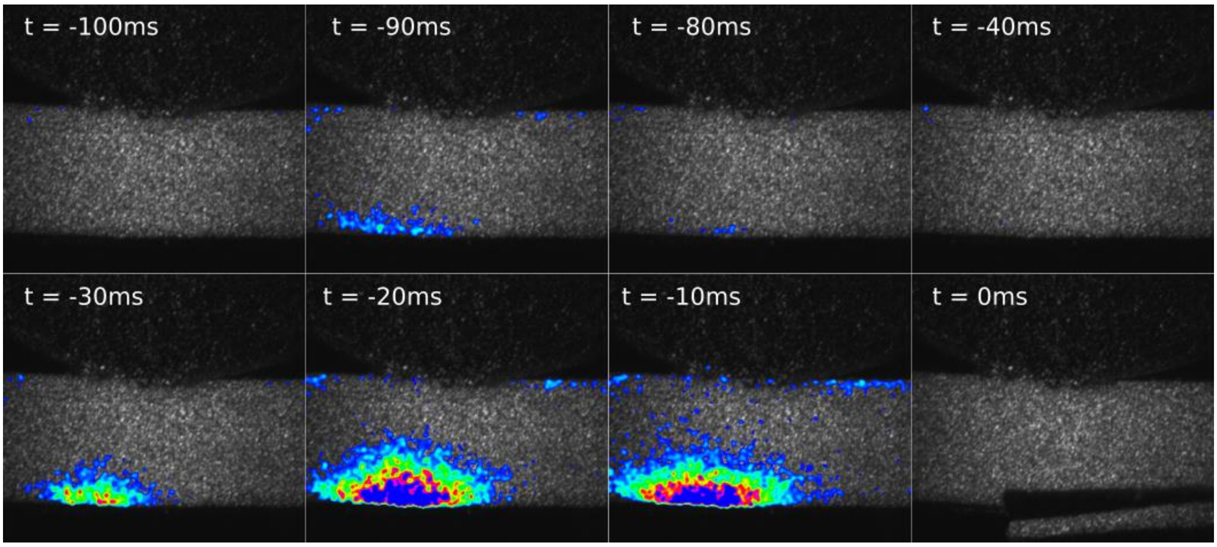

Figure S3. Fracture of an unnotched flexural test sample in laser speckle imaging. First signs of failure could be detected 90 ms before fracture. Just before fracture, a very large damage zone seems to form. The detected events are much faster than in SEB tests, here a $t_{\mathrm{d}} = 10$ µs was used.

Captions for Videos S1-S5:

Video S1. The recorded video data during the experiment on clay/polymer nanocomposite shown in Figure 3. The original data was recorded at 500 s^{-1}, but only every 20th frame is shown in the video.

Video S2. Abalone nacre, the same experiment as shown in Figure 2.

Video S3. Same as Video S1, but with the speckle difference signal overlaid on the video.

Video S4. A different experiment with the nanocomposite.

Video S5. A clay/polymer nanocomposite tested edge-on.
